# Combined Vaccination with B Cell Peptides Targeting Her-2/neu and Immune Checkpoints as Emerging Treatment Option in Cancer

**DOI:** 10.3390/cancers14225678

**Published:** 2022-11-18

**Authors:** Joshua Tobias, Mirjana Drinić, Anna Schmid, Anastasiya Hladik, Martin L. Watzenböck, Claire Battin, Erika Garner-Spitzer, Peter Steinberger, Michael Kundi, Sylvia Knapp, Christoph C. Zielinski, Ursula Wiedermann

**Affiliations:** 1 Center for Pathophysiology, Infectiology and Immunology, Institute of Specific Prophylaxis and Tropical Medicine, Medical University of Vienna, 1090 Vienna, Austria; 2 Department of Medicine I, Research Division of Infection Biology, Medical University of Vienna, 1090 Vienna, Austria; 3 Center for Pathophysiology, Infectiology and Immunology, Division of Immune Receptors and T Cell Activation, Institute of Immunology, Medical University of Vienna, 1090 Vienna, Austria; 4 Department of Environmental Health, Center for Public Health, Medical University of Vienna, 1090 Vienna, Austria; 5 Central European Cancer Center, Wiener Privatklinik and Central European Cooperative Oncology Group (CECOG), 1090 Vienna, Austria

**Keywords:** Her-2/neu, immune checkpoints, combination therapy, vaccination, active immunization, mimotopes/B cell peptides, anti-tumor effect, safety

## Abstract

**Simple Summary:**

Therapies with monoclonal antibodies (mAbs) targeting tumor-associated antigens (TAAs) or immune checkpoint inhibitors (ICIs) have revolutionized cancer treatment. Nevertheless, the inevitable development of resistance and the failure to respond are among this approach’s disadvantages, limiting the duration of disease- or progression-free and overall survival. As an alternative to therapeutically efficacious monoclonal antibodies, the concept of active immunization with vaccines has been repeatedly discussed. In particular, mimotopes, representing the B cell epitope of therapeutic mAbs, have been shown to induce immunological memory and effectively produce antibodies with similar functionality to the respective mAbs/ICIs. This review focuses on a new frontier of vaccinations directed against two cancer-relevant targets, addresses concerns about the safety of active immunization targeting PD-1 and discusses limitations and outlooks.

**Abstract:**

The application of monoclonal antibodies (mAbs), targeting tumor-associated (TAAs) or tumor-specific antigens or immune checkpoints (ICs), has shown tremendous success in cancer therapy. However, the application of mAbs suffers from a series of limitations, including the necessity of frequent administration, the limited duration of clinical response and the emergence of frequently pronounced immune-related adverse events. However, the introduction of mAbs has also resulted in a multitude of novel developments for the treatment of cancers, including vaccinations against various tumor cell-associated epitopes. Here, we reviewed recent clinical trials involving combination therapies with mAbs targeting the PD-1/PD-L1 axis and Her-2/neu, which was chosen as a paradigm for a clinically highly relevant TAA. Our recent findings from murine immunizations against the PD-1 pathway and Her-2/neu with peptides representing the mimotopes/B cell peptides of therapeutic antibodies targeting these molecules are an important focus of the present review. Moreover, concerns regarding the safety of vaccination approaches targeting PD-1, in the context of the continuing immune response, as a result of induced immunological memory, are also addressed. Hence, we describe a new frontier of cancer treatment by active immunization using combined mimotopes/B cell peptides aimed at various targets relevant to cancer biology.

## 1. Introduction

A range of cancer immunotherapy approaches enabling and reactivating the patient’s anti-tumor immune response have shown remarkable advances in recent years [1,2]. The application of therapeutic and tumor-targeting monoclonal antibodies (mAbs), possessing an intrinsic antineoplastic activity, represents the passive form of immunotherapy [3,4,5,6]. Contrarily, anticancer vaccines enhance the activation of the host’s immune system [7,8], referred to as active immunotherapy. Cancer immunotherapy based on the application of mAbs has been successfully established in recent years [9,10,11,12,13]; however, tumor heterogeneity, intra-tumoral factors, the interaction between cancer cells and the immune system, as well as the tumor microenvironment, are amongst factors limiting the efficacy of therapeutically applied monoclonal antibodies [14].

The interaction between T cell receptors and antigens presented in the context of MHC molecules and costimulatory receptors (such as CD28 or CD86) results in the activation of T cells [15]. However, co-inhibitory receptors on T cells result in counterbalanced stimulatory signals. Such co-inhibitory molecules, i.e., immune checkpoints (ICs) include programmed cell death 1 (PD-1), which binds to PD-L1 (B7-H1 or CD274) and PD-L2 (B7-DC or CD273), and cytotoxic T lymphocyte antigen 4 (CTLA-4), which interacts with CD80 or CD86 [16,17,18,19]. Preclinical and clinical evidence has repeatedly demonstrated the ability of tumors to escape immunosurveillance via the expression of surface ligands that engage inhibitory receptors on tumor-specific T cells, thus, resulting in immune tolerance and failure to induce tumor cell death due to T cell anergy/exhaustion [7,20]. Consequently, the blockade of the PD-1/PD-L1 interaction by various mAbs, i.e., immune checkpoint inhibitors (ICIs) [6,21,22,23], which target PD-1 (e.g., nivolumab, pembrolizumab and cemiplimab), PD-L1 (e.g., atezolizumab, avelumab and durvalumab) or CTLA-4 (ipilimumab and tremelimumab) [23,24,25], is considered a milestone in cancer treatment. This has been registered in manifold indications [26,27,28], for example, in the treatment of gastroesophageal cancer [29], hepatocellular carcinoma [30], cervical cancer [31], head and neck cancer [32], urothelial carcinoma [33] and lymphoma [34]. Treatment with ICIs still holds tremendous promise, be it as monotherapy or in combination with either chemotherapy, targeted therapies or other immunomodulatory compounds [35].

**Figure 1:**
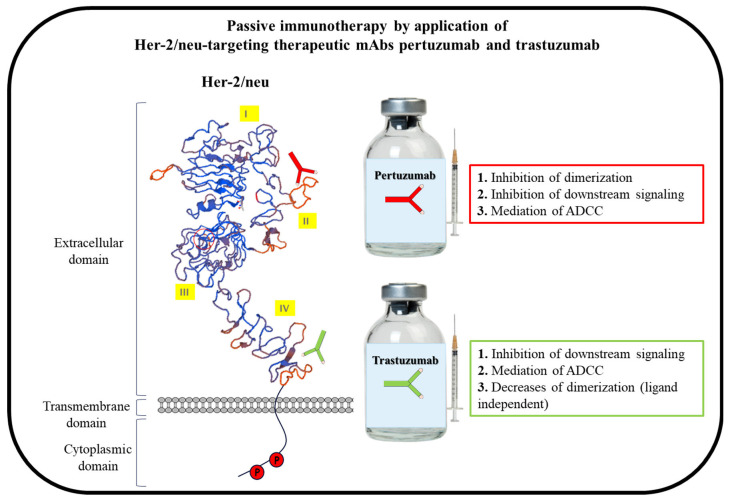
Schematic description of passive immunotherapy with Her-2/neu-targeting therapeutic mAbs pertuzumab and trastuzumab. The mAbs bind to their respective B cell epitopes on extracellular domain II (pertuzumab) and IV (Trastuzumab) of Her-2/neu. The mAbs’ modes of action upon their binding to the receptor are shown in the respective boxes. Adapted with permission from Ref. [62]. Elsevier, 2022.

Her-2/neu, a 185 kDa transmembrane protein, is a member of the human epidermal growth factor receptors (EGFR) family, and its overexpression has been demonstrated in approximately 15–30% of breast and gastric cancers [36,37,38,39,40,41,42]. The receptor is an attractive tumor-associated antigen (TAA) for cancer therapy due to the association of Her-2/neu overexpression with an aggressive biological cancer phenotype and reduced survival in patients with Her-2/neu-positive tumors, as well as improvable response to traditional chemotherapy and, consequently, poor prognosis [42,43,44,45,46]. The extracellular domain of Her-2/neu is divided into four subdomains (I, II, III, IV), with its intracellular domain exhibiting tyrosine kinase activity similar to the other receptors in this family, i.e., Her-1 or EGFR, Her-3 and Her-4 [47,48]. Because no ligand has been identified for Her-2/neu and it is in a constitutively active conformation, the receptor is a preferred partner for dimerization with other members via the dimerization loop located on its extracellular subdomain II. Once paired, the tyrosine residues on the receptor’s intracellular domains are mutually phosphorylated, leading to the initiation of signaling pathways, including the phosphatidylinositol 3-kinase/protein kinase B (PI3K/Akt) pathway, and, consequently, tumor cell proliferation, angiogenesis, invasion and metastasis [49,50,51,52]. By way of different mechanisms, such as antibody-dependent cellular cytotoxicity (ADCC), dimerization inhibition in a ligand-independent manner, the receptor’s degradation and/or internalization and PI3K–AKT signaling pathway inhibition, the first FDA-approved anti-Her-2/neu humanized mAb, trastuzumab, interferes with Her-2/neu signaling [53,54] (Figure 1). The initial therapeutic effect evaluation of trastuzumab showed that treatment with mAb after adjuvant chemotherapy improved overall survival (OS) in women with Her-2/neu-positive metastatic breast cancer [55] and resulted in significantly improved disease-free survival one year after the treatment [56]. As a standard first line of care, trastuzumab is used for the treatment of both early-stage and metastatic Her-2/neu-positive breast cancer. Differing from trastuzumab in its mechanisms of action, pertuzumab, the second FDA-approved anti-Her-2/neu mAb, which is used for the treatment of Her-2/neu-positive metastatic breast cancer, targets the dimerization domain/loop of Her-2/neu and, consequently, results in the inhibition of the receptor’s ligand-induced/dependent dimerization [57,58] (Figure 1). Therefore, by complementary mechanisms of action and in a synergistic manner, which results in a maximal blockade of the Her-2/neu oncogenic pathway [58], the inhibition of tumor growth by the combination of trastuzumab and pertuzumab in both in vitro and in vivo preclinical models has been shown [59,60]. The addition of pertuzumab to trastuzumab and docetaxel was shown in the phase III CLEOPATRA trial to significantly prolong median OS by 15.7 months compared to placebo plus trastuzumab and docetaxel [61].

ICIs have been proposed to strengthen an emerging immune response following vaccination against TAAs. Here, we summarize the latest reports on the application of Her-2/neu-targeting mAbs and ICIs and vaccination strategies for targeting Her-2/neu and immune checkpoints.

## 2. Combination of Her-2/neu-Targeted Therapy with Immune Checkpoint Blockade

### 2.1. Basic Considerations

Trastuzumab’s capacity in upregulating PD-L1 expression, by the recruitment of IFNgamma-secreting immune effector cells, has been shown as a mechanism of resistance to trastuzumab [63]. Furthermore, the mAbs that are bound to tumor cells engage innate immune effector cells via their Fc receptor, resulting in ADCC [64,65], which in turn also results in the upregulation of PD-L1 [66]. It has been shown that the increased ability of tumor cells to evade the immune system is attributed to their PD-L1 expression capacity, allowing their interaction with PD-1-expressing immune cells [67]. Because the application of ICIs aims to inhibit the interaction of PD-1 with PD-L1 and increase T cell survival and proliferation, consequently leading to an enhanced tumor-directed immune response, it has been suggested that combining ICIs with anti-Her-2/neu mAbs, such as Trastuzumab, could act in a synergistic manner to delay or even prevent resistance to the mAb [68]. Thus, the progression of Her-2/neu-positive metastatic breast cancer, as a result of primary or secondary resistance to Her-2/neu-targeted therapies with trastuzumab [69], might be hindered by combining with an immune checkpoint blockade [70,71].

### 2.2. Clinical Trials for Combination Therapies of Her-2/neu-Targeting Compounds plus ICIs

A phase II trial (ClinicalTrials.gov Identifier: NCT02129556) enrolled 52 patients with advanced Her-2/neu-positive breast cancer who had progressed on trastuzumab treatment [72] (Table 1). Patients received anti-PD-1 mAb pembrolizumab and standard trastuzumab. Patients with PD-L1-positive tumors demonstrated a disease control rate of 25% (90% CI, 14–39), with a 12-month progression-free survival (PFS) and OS of 12% and 65%, respectively, whereas patients with PD-L1-negative tumors did not experience a benefit from such treatment. The results from this trial were promising and indicated that Her-2/neu-positive PD-L1 overexpressing breast cancer patients benefited from anti-PD-1 therapy [72]. In a recently completed randomized phase II trial (ClinicalTrials.gov Identifier: NCT02924883), the addition of anti-PD-L1 mAb atezolizumab to T-DM1 (trastuzumab covalently linked to the cytotoxic agent DM1) [73] was evaluated in patients with unresectable or metastatic Her-2/neu-positive breast cancer who had received prior trastuzumab- and taxane-based therapy [74] (Table 1). Patients with PD-L1-positive breast cancers from the atezolizumab group had longer PFS than those with PD-L1-negative tumors. Furthermore, patients in the atezolizumab group whose tumors had ≥ 5% infiltrating-tumor lymphocytes (TILs) had longer PFS than patients with a TILs of <5% [74]. On the basis of the results of this trial, a randomized phase III trial (ClinicalTrials.gov Identifier: NCT04873362; Table 1) is now being conducted to evaluate T-DM1 ± atezolizumab in PD-L1 overexpressing breast cancers with residual invasive Her-2/neu-positive breast cancer following neoadjuvant taxane-based and Her-2/neu-targeted therapy.

As also shown in the case of other types of cancer [75], patients with PD-L1-positive tumors may potentially benefit more from PD-1 or PD-L1 inhibition than those with PD-L1-negative tumors. Ongoing trials aim to evaluate PD-1-, PD-L1- or CTLA4-targeting ICIs in combination with standard anti-Her-2/neu therapy for Her-2/neu-positive breast cancer (Table 1).

**Table 1:** List of main completed/active/recruiting clinical trials with ICIs and anti-Her-2/neu-targeted therapies, with or without combination with biological drugs.

Evaluated Drugs			Condition/Disease	Phase	Status	NCT/ Trial’s Identifier	Setting	Reference
Anti-Her-2 mAb	ICI	Biological/ Other Drug						
-	Avelumab	Taxane and anthracycline	Metastatic or locally advanced solid tumors	I	Completed	NCT01772004	Dose escalation trial	[76]
Trastuzumab, pertuzumab and trastuzumab emtansine	Atezolizumab	Carboplatin, docetaxel, doxorubicin and cyclophosphamide	Her-2/neu-positive and negative metastatic breast cancer and locally advanced early breast cancer	I	Completed	NCT02605915	Two cohorts with several arms, which evaluated the different combinations of the examined drugs	[77]
Trastuzumab	Pembrolizumab		Advanced, trastuzumab-resistant Her-2/neu-positive metastatic breast cancer	Ib, II	Completed	NCT02129556 (PANACEA)	trastuzumab with pembrolizumab	[72]
Trastuzumab emtansine	Atezolizumab		Her-2/neu-positive locally advanced or metastatic breast cancer	II	Completed	NCT02924883	Arm 1: trastuzumab emtansine and placebo Arm 2: trastuzumab emtansine and atezolizumab	[74]
Trastuzumab and pertuzumab	Atezolizumab		Her-2/neu-positive metastatic breast cancer that has spread to the brain	II	Active	NCT03417544	Arm: trastuzumab, pertuzumab and atezolizumab	Clinicaltrials.gov
Trastuzumab and pertuzumab	Atezolizumab	Doxorubicin, cyclophosphamid and paclitaxel	Early Her-2/neu-positive breast cancer	III	Active	NCT03726879	Arm 1: placebo, doxorubicin, cyclophosphamide, paclitaxel, trastuzumab, pertuzumab and trastuzumab emtansine Arm 2: atezolizumab, doxorubicin, cyclophosphamide, paclitaxel, trastuzumab, pertuzumab and trastuzumab emtansine	Clinicaltrials.gov
Trastuzumab and pertuzumab	Atezolizumab	Taxane, paclitaxel and docetaxel	Neoadjuvant treatment of Her-2/neu-positive early high-risk and locally advanced breast cancer	III	Active	NCT03595592	Arm 1: trastuzumab, pertuzumab, paclitaxel and carboplatin Arm 2: trastuzumab, pertuzumab, paclitaxel, carboplatin, doxorubicin, cyclophosphamide and atezolizumab Arm 3: trastuzumab, pertuzumab, paclitaxel, carboplatin and atezolizumab	Clinicaltrials.gov
Trastuzumab and pertuzumab	Atezolizumab	Taxane, paclitaxel and docetaxel	Her-2/neu-positive metastatic breast cancer	III	Active	NCT03199885	Arm 1: trastuzumab, pertuzumab, taxane therapy and placebo Arm 2: trastuzumab, pertuzumab, taxane therapy and atezolizumab	Clinicaltrials.gov
Trastuzumab deruxtecan (T-DXd), trastuzumab and pertuzumab	Durvalumab	Deruxtecan, paclitaxel and tucatinib	Her-2/neu-positive metastatic breast cancer, without (part 1) or with brain metastases (part 2)	I, II	Recruiting	NCT04538742	Part 1: Arm 1: T-DXd Arm 2: T-DXd and durvalumab Arm 3: T-DXd and pertuzumab Arm 4: T-DXd and paclitaxel Arm 5: T-DXd and durvalumab and paclitaxel Arm 6: T-DXd and tucatinib Part 2: Arm 7: T-DXd Arm 8: T-DXd and tucatinib	Clinicaltrials.gov
VRP-Her-2/neu and pembrolizumab			Patients with Her-2/neu breast cancer	II	Recruiting	NCT03632941	Arm 1: VRP-Her-2/neu vaccine Arm 2: pembrolizumab Arm 3: VRP-Her-2/neu vaccine and pembrolizumab	[78]
Trastuzumab	Atezolizumab	Vinorelbine	Her-2/neu-positive advanced/ metastatic breast cancer	II	Recruiting	NCT04759248	Arm: trastuzumab, atezolizumab and vinorelbine	Clinicaltrials.gov
Trastuzumab	Tremelimumab and durvalumab		Her-2/neu-positive metastatic breast cancer	II	Recruiting	BCT 1703	Arm: durvalumab, tremelimumab and trastuzumab	breastcancertrials.org.au
Trastuzumab	Avelumab and utomilumab [79]	Vinorelbine	Advanced Her-2/neu-positive breast cancer	II	Recruiting	NCT03414658	Arm 1: trastuzumab and vinorelbine Arm 2: trastuzumab, vinorelbine and avelumab Arm 3: trastuzumab, vinorelbine, avelumab and utomilumab Arm 4: trastuzumab, avelumab and utomilumab	Clinicaltrials.gov
Trastuzumab and pertuzumab	Pembrolizumab	Paclitaxel	Chemo naive patients with invasive Her-2/neu-positive breast cancer	II	Recruiting	NCT03747120	Arm 1: trastuzumab, pertuzumab and paclitaxel Arm 2: trastuzumab, pertuzumab, pembrolizumab and paclitaxel Arm 3: trastuzumab, pembrolizumab and paclitaxel	Clinicaltrials.gov
Trastuzumab emtansine	Atezolizumab		Her-2/neu-positive breast cancer at high risk of recurrence following preoperative therapy	III	Recruiting	NCT04873362	Arm 1: placebo and trastuzumab emtansine Arm 2: atezolizumab and trastuzumab emtansine	Clinicaltrials.gov
Trastuzumab emtansine	Atezolizumab		Her-2/neu-positive and PD-L1-positive locally advanced or metastatic breast cancer	III	Recruiting	NCT04740918	Arm 1: T-DM1 and placebo Arm 2: T-DM1 and atezolizumab	Clinicaltrials.gov
Trastuzumab emtansine	Atezolizumab		Her-2/neu-positive primary breast cancer	III	Recruiting	NCT04873362	Arm 1: trastuzumab emtansine and placebo Arm 2: trastuzumab emtansine and atezolizumab	Clinicaltrials.gov

ICI: Immune Checkpoint Inhibitor; and VRP-Her-2/neu: virus-like replicon particles (VRP) packaged with an alphaviral vector encoding the extracellular domain and transmembrane regions of Her-2/neu.

### 2.3. B Cell Peptides/Mimotopes-Based Vaccination Targeting Her-2/neu and ICs

Despite tremendous therapeutic success with the use of mAbs, their continuous application over a long period, their half-life [80] and lack of capacity to induce immunological memory [81] may limit the duration of therapy, resulting in only temporary disease control, in particular, once the tumor has metastasized [82,83,84,85,86,87,88,89]. Further drawbacks of treatments with mAbs include the high frequencies of non-responsiveness [90], the development of resistance [91], and immune-related adverse events and hypersensitivity to treatments with mAbs [92,93], possibly due to the high doses of mAbs that ensure their immediate therapeutic effect [94,95]. In contrast, vaccines based on TAAs or mimotopes representing therapeutic mAb binding epitopes can induce prolonged activation of the immune system and mount immunological memory, which, in association with booster vaccinations, potentially results in tumor involution [96].

**Figure 2:**
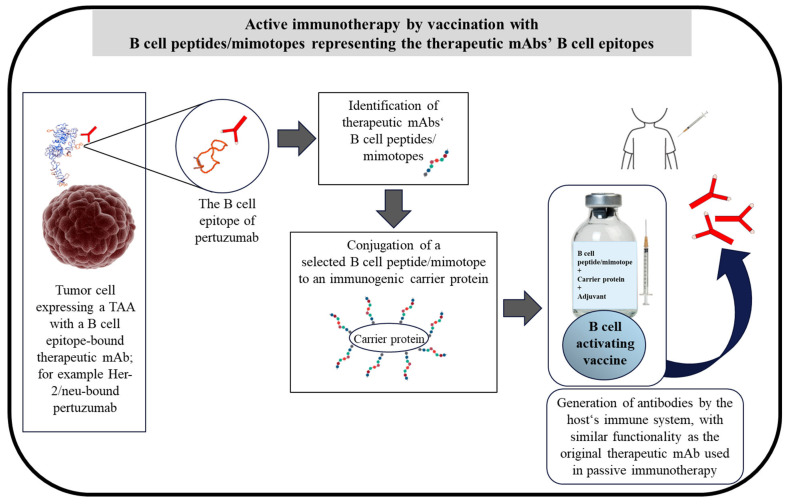
Schematic description of active immunotherapy with B cell peptides/mimotopes representing the B cell epitopes of therapeutic mAbs on TAAs, for example, a B cell peptide/mimotope of pertuzumab. The binding epitope of pertuzumab indicates the mAb B cell epitope on Her-2/neu. B cell peptides/mimotopes representing the mAb binding epitopes are identified, as described in the text and previously [62,101,102,103]. The selected B cell peptide/mimotope of the mAbs is conjugated to an immunogenic carrier protein, and an admixture-based vaccine comprising the conjugate and an adjuvant is then formulated. The formulated vaccine directly activates B cells for the production of antibodies in the host. The production of the antibodies is also induced further by the presence of T cell epitopes in the carrier protein, resulting in stimulation of T cells and further activation of B cells. The endogenously produced antibodies have the same functionality and modes of action as the respective therapeutic mAbs, such as pertuzumab. Adapted with permission from Ref. [62]. Elsevier, 2022.

Mimotopes are peptides that mimic and represent the immunodominant epitopes on a target protein (TAA or tumor-specific antigens) or the binding epitopes of therapeutic mAbs [97]. Mimotopes identified by antibodies or therapeutic mAbs are solely a representation of the antibodies’ B cell epitopes and, thus, are considered as B cell peptides. Such mimotopes, consequently, not only inhibit the binding of the antibodies to the respective antigen or protein but also, upon conjugation to an immunogenic carrier protein, induce an epitope-specific antibody response. As depicted in Figure 1, the application of mAbs represents the passive immunotherapy approach, while vaccination with B cell peptides/mimotopes is the active immunotherapy approach (Figure 2). Endogenously generated antibodies after vaccination can induce anti-tumor responses for prolonged periods of time by the induction of immunological memory [98].

The identification of mimotopes can be based on the use of phase display strategies [99,100] or, alternatively, as applied in our group, by computer algorithms or the use of synthesized overlapping bio-peptides from the sequence of the respective protein [62,101,102,103]. The peptides are screened and the mimotope candidates are selected based on their capacity to inhibit the binding of the examined mAb to the respective protein [62,101,102,104]. For the evaluation of immunogenicity by vaccination strategy and the anti-tumor effect, the mimotopes (B cell peptides) are conjugated to a carrier protein and administered together with an adjuvant [62,101,102,104] (Figure 2). Mimotopes have demonstrated a promising approach in the field of allergy [105,106], infectious diseases [107,108,109] and for cancer therapy, by inducing an anti-tumor effect targeting PD-1 [101], and Her-2/neu-expressing solid tumors [62,100] and lung metastasis [62,102]. The use of overlapping peptides as mimotopes with T cell immunodominant epitopes has also been evaluated in a phase I immunotherapeutic trial against human papillomavirus 16, and shown tolerability and the induction of the T cell response, even in the patients with end-stage disease [110].

#### 2.3.1. Her-2/neu B Cell Peptide/Mimotope

In line with active immunization/vaccination with B cell peptides [8,62,101,102,103], we have formulated the anti-Her-2/neu vaccine HER-Vaxx [111] consisting of a hybrid peptide (P467) [111,112]. The cross-reacting material CRM197, a mutated and nontoxic form of diphtheria toxin [113] with the capacity to activate B cells and CD4+ T cells with a heterogeneous Th1 and Th2 cytokine profile [114], is used as a carrier protein conjugated to the peptide, and the conjugate is administered with the Th1/Th2-driving adjuvant Montanide (ISA-51-VG) [62,102,103,111,115,116]. The Her-2/neu-directed B cell peptide-based vaccine HER-Vaxx has been evaluated in an array of preclinical experiments [102,111,112,117]. In phase 1b and Phase II trials involving patients suffering from Her-2/neu overexpressing metastatic or advanced adenocarcinoma of the stomach the vaccine was shown to be safe, immunogenic, and to prolong progression-free survival [118,119,120].

Given that, in Her-2/neu-positive metastatic breast cancer patients, the combined treatment of trastuzumab with pertuzumab has established an incredible achievement, and to broaden the binding spectrum of the induced antibodies by our vaccine, we recently constructed a multi-peptide B cell vaccine comprising HER-Vaxx and pertuzumab’s mimotope (HSGICELHCPALVTYNTDTFESMPNPEGRYTFGASCVTACPY; amino acids (AAs) 260–301, Uniprot P04626) and tested its capacity to prevent the formation of metastases in a mouse model with Her-2/neu lung metastases [62,102]. Active immunization with the multi-peptide vaccine combining HER-Vaxx and the mimotope of pertuzumab resulted in a significant reduction of lung metastasis formation, reflected by the reduction of lung weight and the size of the metastases, which was shown to be prominently associated with the formation of Her-2/neu-negative tumors with increased PD-L1 expression [102]. Because ICIs have been proposed to strengthen an emerging immune response following vaccination against TAAs, this observation suggests that a combination of a multi-peptide B cell Her-2/neu vaccine together with anti-PD-L1 ICI or active immunization with a mimotope from PD-L1 might serve as a suitable intervention to prevent metastasis formation.

#### 2.3.2. PD-L1 B Cell Peptide/Mimotope

**Figure 3:**
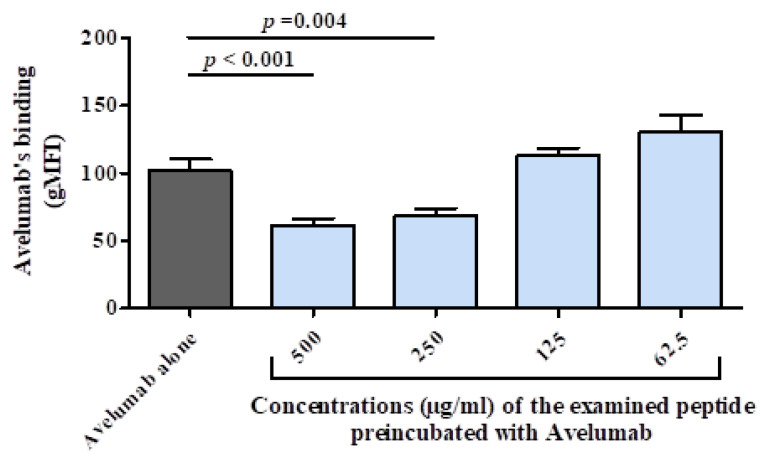
Examination of the ‘hybrid hPDL1-mimotope’ in cellular assay. Jurkat T cells expressing hPD-L1 were used in a cellular assay to assess the binding of avelumab (30 ng/mL) alone or after pre-incubation with different concentrations of the examined peptide. The values represent the mean and standard deviation of the geometric mean of the fluorescence intensity (gMFI) of the viable population of the cells from three independent experiments. Significant differences are indicated by the respective *p*-values.

We recently used avelumab as one of the therapeutic mAbs targeting PD-L1 to identify its mimotope. On the basis of the crystal structure of human PD-L1 (hPD-L1) complexed with the single-chain Fv fragment of avelumab, it has been shown that the mAb binds to the IgV domain of the immune checkpoint [121]. By applying our platform for the identification of avelumab mimotope [101], 20-mer overlapping peptides spanning the IgV domain of hPD-L1 were tested and two B cell epitopes (EKQLDLAALIVYWEMEDKNIIQFVH, AAs 45-69; VYRCMISYGGADYKR, AAs 111–125; Uniprot Q9NZQ7) were selected. The peptide ‘hybrid hPDL1-mimotope’ consisting of the two mimotopes linked with a flexible glycine linker (GGGG) was generated. A cellular binding assay [122] using Jurkat reporter cells expressing hPD-L1 was employed to examine the inhibitory capacity of the hybrid peptide. As shown in Figure 3, avelumab alone could effectively bind to hPD-L1-expressing Jurkat cells. However, pre-incubation of the mAb with the peptide dose-dependently inhibited the binding of the mAb. On the basis of these data, in vivo investigations are ongoing to examine the effect of vaccination/active immunization with the hybrid peptide on inducing anti-tumor effects in a mouse model with syngeneic tumors expressing Her-2/neu [101].

#### 2.3.3. PD-1 B Cell Peptide/Mimotope

An alternative approach to target the PD-1/PD-L1 axis and block their interaction is by targeting PD-1. The sequences of human PD-1 (hPD-1) and mouse PD-1 (mPD-1) were used to identify the mimotopes JT-N1 (PGWFLDSPDRPWNPP; AAs 21–35, Uniprot Q15116) and JT-mPD1 (ISLHPKAKIEESPGA; AAs 126–140, Uniprot Q02242), respectively, and we have presented, for the first time, the concept of targeting PD-1 by vaccination using mimotopes [101,103]. A strong induced anti-tumor effect in vivo, to an extent similar to the corresponding mAb, was shown after active immunization with the mimotope [101]. The combination of the mimotope JT-mPD1 with the anti-Her-2/neu vaccine HER-Vaxx [111,120] led to an increase in the HER-Vaxx-generated anti-tumor effect [101]. In that manner, a strong anti-tumor effect in vivo was also shown by a peptide (PD-1-Vaxx) residing at the position 92–101 of hPD-1 [123]; the safety, tolerability and immunogenicity of the peptide as monotherapy in patients with PD-L1 expressing non-small cell lung cancer (NSCLC) is being evaluated in a phase I clinical trial [124].

The use of ICIs is often hampered by immune-related adverse events (irAEs) and hypersensitivity [92,93,125,126]. As mentioned above, vaccination with mimotopes/B cell peptides results in immunological memory. With this approach, targeting PD-1 or PD-L1 may result in the continuous inhibition of the PD-1/PD-L1 interaction and, thus, increased irAEs. Therefore, we were prompted to evaluate the safety of this approach in an influenza infection mouse model, as described in the next section.

#### 2.3.4. Safety of B Cell Peptide/Mimotope-Based Vaccination Targeting PD-1

In 2018, a small study reported an unexpectedly high incidence (52% of 23 patients) of irAEs in influenza-vaccinated patients receiving anti-PD-1 inhibitors [127]. However, in a larger study with 127 lung cancer patients receiving nivolumab therapy, 47 patients who received vaccinations against influenza showed no difference in incidence or severity of irAEs [128]. In a retrospective study involving 370 patients receiving ICI therapy (nivolumab and pembrolizumab), 20% experienced an irAE of any grade [129]. These results were confirmed by a follow-up study [130]. Furthermore, in a small study involving 24 patients treated with anti-PD-1 or PD-L1 mAbs, the majority of the irAEs following vaccination with inactivated influenza vaccine were graded 1–2, therefore not requiring a change of ICI therapy [131]. Taken together, these results did not raise safety concerns regarding the application of an influenza vaccination in combination with ICI treatment. Nevertheless, we made use of the described observations and examined whether active immunization with a mimotope targeting PD-1 could affect the antiviral immune response. As described in Figure S1, an influenza infection model was established in BALB/c mice. A selected dose of 50 plaque-forming units (PFU) was used to examine whether active immunization with the mimotope from mouse PD-1 or treatment with the corresponding mAb (ICI) enhanced the antiviral cellular immune response. Following active immunization with the mimotope (JT-mPD1) or, for comparison, after the application of a functional anti-mouse PD-1 mAb, which was used for the identification of the mimotope [101], mice were infected with the influenza virus strain (A/PR/8/34) and sacrificed on days five and nine post-infection for clinical and immunological evaluations (Figure S2A).

**Figure 4:**
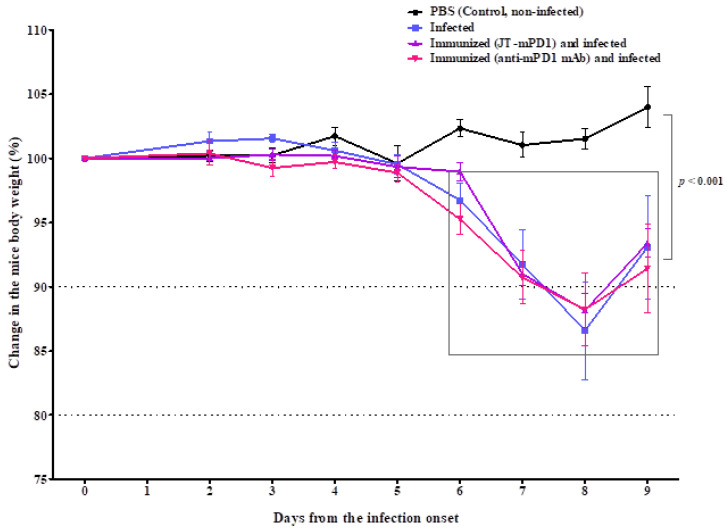
On the day of the infection (day 0) and each day after the infection, the body weight of the mice was measured. Each point indicates an average of 5 mice, shown with SE. Significant differences are indicated by the respective *p*-values.

As shown in Figure 4, the viral infection resulted in a significant decrease in body weight in all the infected mice around the peak day (day eight) of the infection. However, the observed body weight reduction was similar in all the infected groups, indicating that neither active immunizations with the mimotope nor the application of the corresponding mAb were associated with increased disease severity.

The level of viral load in the lungs of the infected mice was assessed based on the mRNA level of the influenza A virus matrix protein gene (M) and showed no significant difference between the viral loads in the infected groups of mice (Figure S2B).

Active immunization with the mimotope induced PD-1-specific serum IgG antibody response in the immunized mice, detected on days five and nine post-infection (Figure S2C). An induction of PD-1-specific serum IgG antibody response was also observed on day nine but not on day five post-infection in mice treated with the mAb (a rat anti-mouse PD-1 antibody) (Figure S2C). A similar observation was shown in clinical settings with patients treated with Nivolumab or Pembrolizumab [132], which might be attributed to a gradual induction of serum Abs against secreted soluble PD-1 as a consequence of the anti-PD-1 treatment. The evaluation of lymphoid (Figure S3A,B) and myeloid (Figure S4) cell populations, the mRNA level of the Th1 cytokine IFNgamma and the pro-inflammatory cytokines IL-6 and TNF (Figure S5) showed no significant difference between the infected mice, which were either untreated, actively immunized with the mimotope from PD-1 or treated with an anti-mPD-1 mAb. Overall, these findings suggest that active immunization with the mimotope is safe and is not associated with increased inflammatory responses, i.e., increased weight loss, an elevated influx of inflammatory immune cells or higher levels of pro-inflammatory cytokines.

## 3. Further Compounds for Targeting Her-2/neu and ICs by Active and Passive Immunotherapies

As described above, the Her-2/neu-directed trastuzumab and pertuzumab have shown a tremendous effect in the clinic. However, the resistance to treatment with such mAbs has resulted in the development of additional mAbs. Increased trastuzumab-mediated ADCC has been shown as a result of polymorphism in the Fc receptors (FcγRs) of IgGs expressed on cytotoxic cells, affecting the ADDC capacity of the respective mAbs [133]. Accordingly, a chimeric anti-Her-2 mAb, margetuximab (MGAH22), with an Fc domain modification for improved binding to FcγRIIIa was constructed [134]. On the basis of a first-in-human phase 1 study of margetuximab, in patients with Her-2/neu-positive advanced solid tumors, the mAb exhibited safety and an anti-tumor effect [135], and, in a phase 3 trial, the treatment with the mAb was associated with improved clinical outcomes in FcγRIIIa 158F allele carriers [136]. An FDA-approved trastuzumab-based drug conjugate, as a second-line therapy for the treatment of Her-2/neu-positive metastatic breast cancer patients with disease progression, is T-DM1 (Kadcyla®, Roche Pharma AG, Grenzach-Wyhlen, Germany), which is comprised of the mAb conjugated to DM1 (emtansine) [137,138]. In addition to retaining the ADCC activity of the mAb, the drug results in a cytotoxic effect by the delivered microtubule-inhibitory agent DM1 (derivative of maytansine) [137].

The tremendous success of the application of ICIs is also evident, as described above and by others [139,140,141]. However, the development of irAEs, fatal toxicities and resistance are the significant drawbacks of treatments with ICIs therapy [142,143], with the latter drawback often associated with decreased or loss of neoantigens immunogenicity, increased levels of immunosuppressive immune cells and also the upregulation of other ICs [144]. Therefore, targeting newer ICs associated with tumor microenvironment is a strategy to overcome the limitations of ICIs [145]. Such new ICs include lymphocyte activation gene-3 (LAG-3) [146], T cell immunoglobulin and ITIM domain (TIGIT) [147], T cell immunoglobulin and mucin-domain containing-3 (TIM-3) [148], V-domain immunoglobulin suppressor of T cell activation (VISTA) [149,150,151], B7 homolog 3 protein (B7-H3) [152] and inducible T cell costimulatory (ICOS) [153]. These ICs represent promising options for treating solid tumors, with clinical trials currently under active investigation for evaluating their effectiveness as monotherapy or combination therapy together with other ICIs [145]. An additional approach for combatting resistance to ICs treatment is by the use of bispecific antibodies [154,155]. FS118 is a novel, tetravalent (bispecific) antibody targeting LAG-3 and PD-L1 [156]. Treatment with the mAb resulted in a decreased expression of LAG-3 on T cells, and it was shed from the target cells, whereas the expression of the IC increased following treatment involving a combination of mAbs singularly targeting LAG-3 and PD-L1 [156]. A durable response to the mAb in a patient with anaplastic thyroid carcinoma who had progressed after PD-1 monotherapy was recently reported [157].

In line with the active immunotherapy/vaccination approach against Her-2/neu and ICs, the application of computer-aided analyses and X-ray structures of mAbs-bound proteins led to the identification of two B cell epitopes representing trastuzumab-binding and pertuzumab-binding epitopes [158,159]. A vaccine (B-Vaxx) comprising the two epitopes [158,159] was shown to be safe and immunogenic in a phase 1 trial [160]. With a similar approach, a vaccine (PD1-Vaxx) with a PD-1 B cell peptide linked to a measles virus fusion peptide via a four amino acid residue (GPSL) was constructed [160] and demonstrated synergistic vaccine combinations with a Her-2-targeted vaccine (B-Vaxx) [123]. Applying the same strategy, a PD-L1 B cell peptide vaccine (PDL1-Vaxx) was also constructed and in combination with a dual Her-2 B cell vaccine (B-Vaxx) was recently shown to induce potent immune responses and effective anti-tumor immunity in multiple syngeneic mice models [161]. In line with these results and our earlier findings showing that targeting Her-2/neu results in the loss of the receptor [102,120] associated with the upregulation of PD-L1 expression [102], two clinical trials were planned (Imugene Limited, Sydney, Australia) to assess: the combination of HER-Vaxx with chemotherapy or with anti-PD-1 antibody pembrolizumab in patients with Her-2/neu overexpressing gastric cancer who have failed treatment with trastuzumab and the combination of HER-Vaxx with chemotherapy ± the anti-PD-L1 antibody avelumab in patients with Her-2/neu overexpressing gastric cancer.

## 4. Conclusions

The downregulation of T cell activation by the PD-1/PD-L1 axis, which is necessary for peripheral tolerance, can be exploited by tumor cells, resulting in the induction of an immunosuppressive state and their growth and immune escape. The application of therapeutic vaccines, based on immunogenic peptides inducing the production of antibodies in the host with a functionality similar to ICIs in inhibiting the PD-1/PD-L1 interaction, is a promising approach for immunotherapy. Additionally, such an approach might potentially overcome some of the disadvantages of the therapeutic mAbs as discussed in this review. A continuous inhibition of the PD-1/PD-L1 interaction may result from a peptide-based vaccination, consequently leading to immunological memory and elevated T cell activity. However, the results of our investigations did not indicate any safety concerns regarding the targeting of immune checkpoint PD-1 by active immunization, nor showed association with the hyper induction of pro-inflammatory cytokine production, known as ‘cytokine storm’ [162]. Nevertheless, the evaluation of the safety, immunogenicity and tolerability of vaccination with a PD-1 peptide (PD-1-Vaxx) is the aim of the ongoing phase 1 clinical trial (ClinicalTrials.gov Identifier: NCT04432207).

On the basis of the findings reviewed here, combined vaccination concepts co-targeting Her-2/neu and the PD-1 pathway seem to present a new treatment strategy to overcome mAbs above-mentioned associated disadvantages. These aspects are currently the topic of ongoing clinical trials, which will shed more light on the clinical outcomes of the discussed approach.

## Data Availability

The data presented in this study are available in the article and supplementary materials.

## Supplementary Materials

The following supporting information can be downloaded at: https://www.mdpi.com/article/10.3390/cancers14225678/s1, Figure S1: Established influenza infection model in BALB/c mice infected with 50PFU of mouse-adapted influenza A/PR/8/34 virus, Figure S2: Immunization schedule, levels of mRNA expression and levels of antibodies, Figure S3: Distribution of immune cell profiles, Figure S4: Distribution of myeloid cell profiles in the examined mice measured by flow cytometry in lungs on day 5 and 9 after infection, Figure S5: The levels of mRNA expression of IFNgamma, IL-6 and TNF in lungs, measured by RT-PCR.
