# Supplementary material for: Combined Vaccination with B Cell Peptides Targeting Her-2/neu and Immune Checkpoints as Emerging Treatment Option in Cancer
Source: Cancers (Basel). 2022 Nov 18;14(22):5678. doi: 10.3390/cancers14225678 (PMC9688220; doi:10.3390/cancers14225678)
Supplement: Supplementary file 1 [file cancers-14-05678-s001.zip › cancers-1985339-supplementary.pdf]

cancers MDPI

Supplementary Materials: Combined Vaccination with B Cell Peptides Targeting Her-2/neu and Immune Checkpoints as Emerging Treatment Option in Cancer

Joshua Tobias [1,*], Mirjana Drinić [1], Anna Schmid [1], Anastasiya Hladik [2], Martin L. Watzenböck [2], Claire Battin [3], Erika Garner-Spitzer [1], Peter Steinberger [3], Michael Kundi [4], Sylvia Knapp [2], Christoph C. Zielinski [5] and Ursula Wiedermann [1,*]

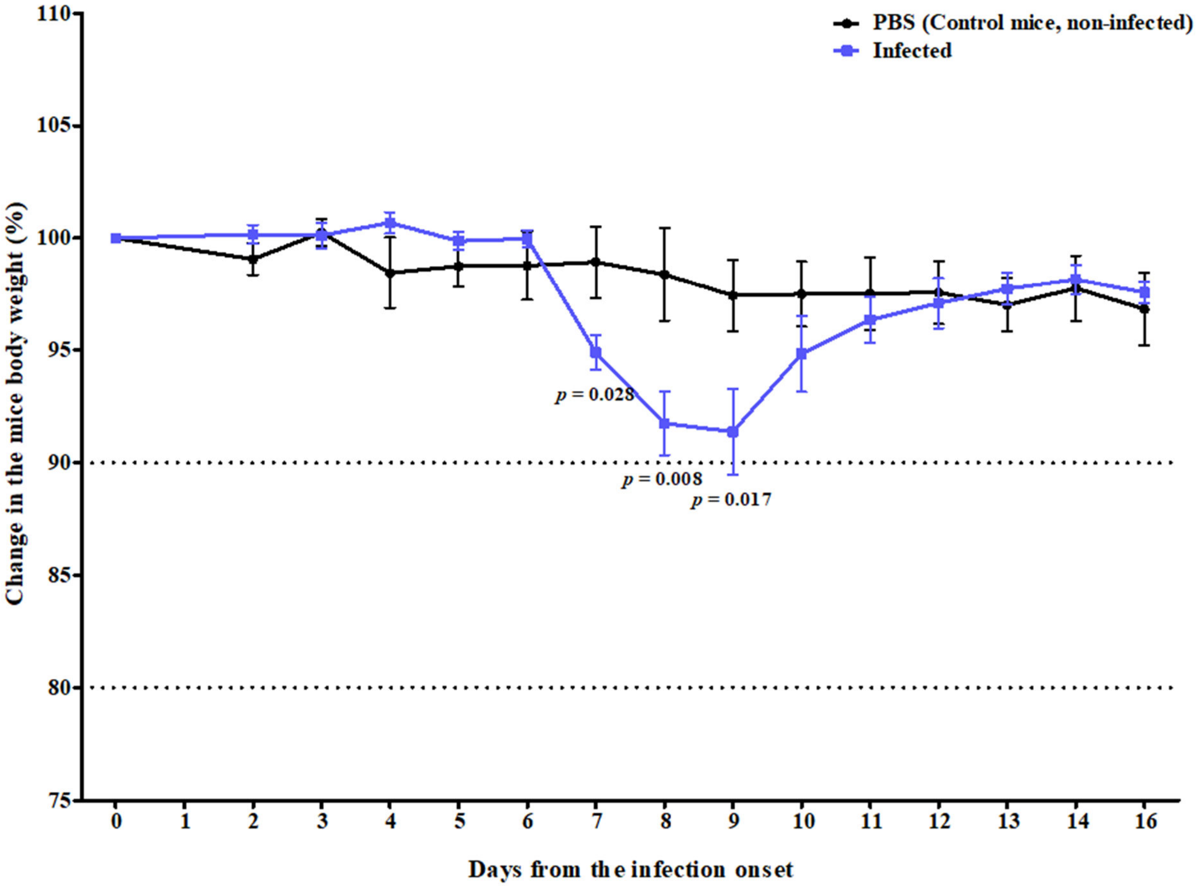

Figure S1. Established influenza infection model in BALB/c mice infected with 50PFU of mouse-adapted influenza A/PR/8/34 virus.

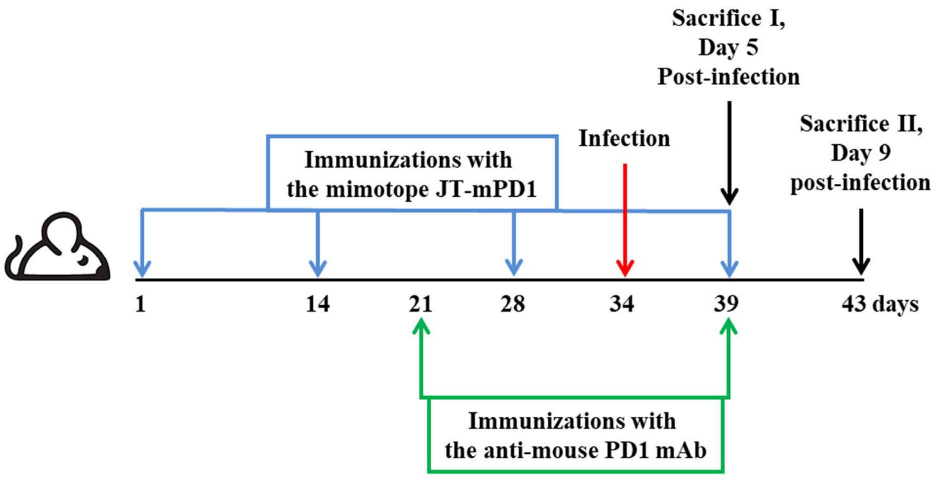

A

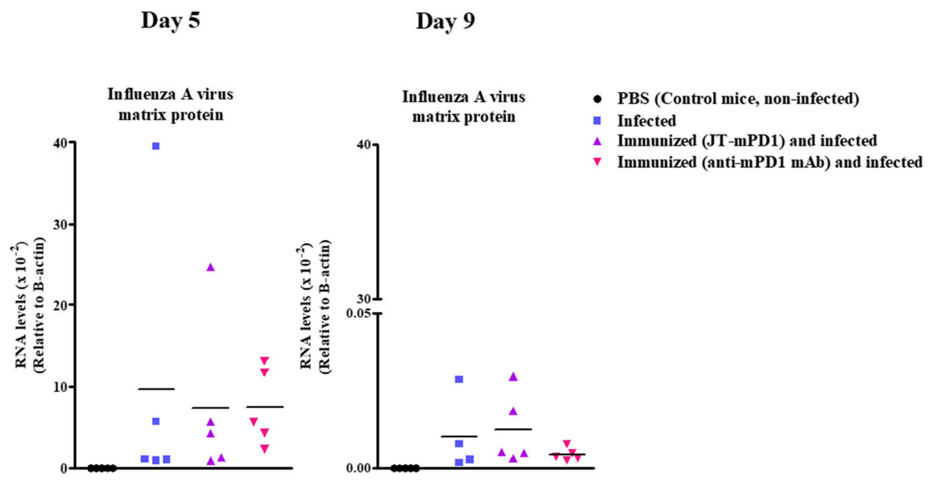

B

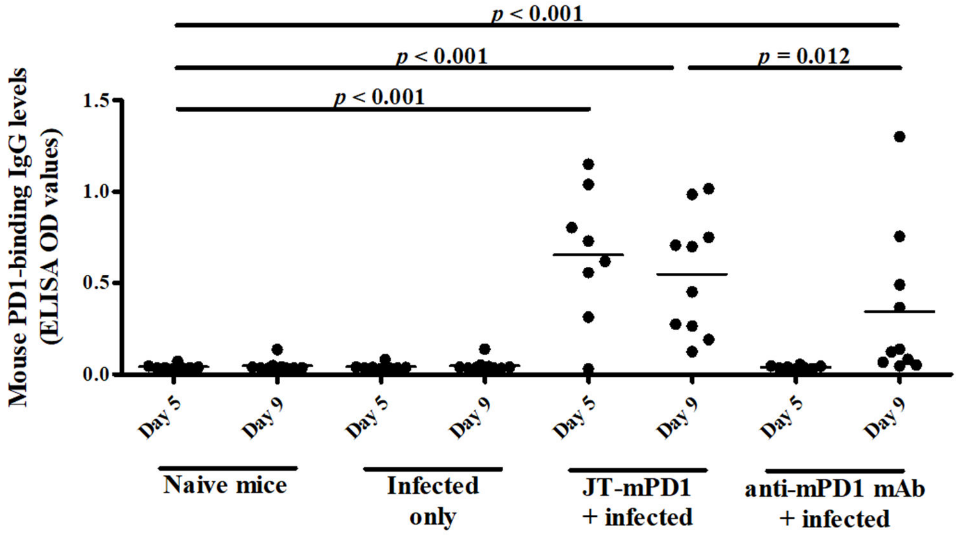

C

Figure S2. Immunization schedule, levels of mRNA expression and levels of antibodies. (**A**) immunization schedule for the evaluation of the effect of active immunization with the mimotope of mPD-1 (JT-mPD1) or passive immunization with the respective functional anti-mPD-1 mAb; (**B**) the levels of mRNA expression of Influenza matrix in lungs, measured by RT-PCR on day 5 and 9 after infection; (**C**) the levels of antibodies against mouse PD1 induced in the immunized mic.

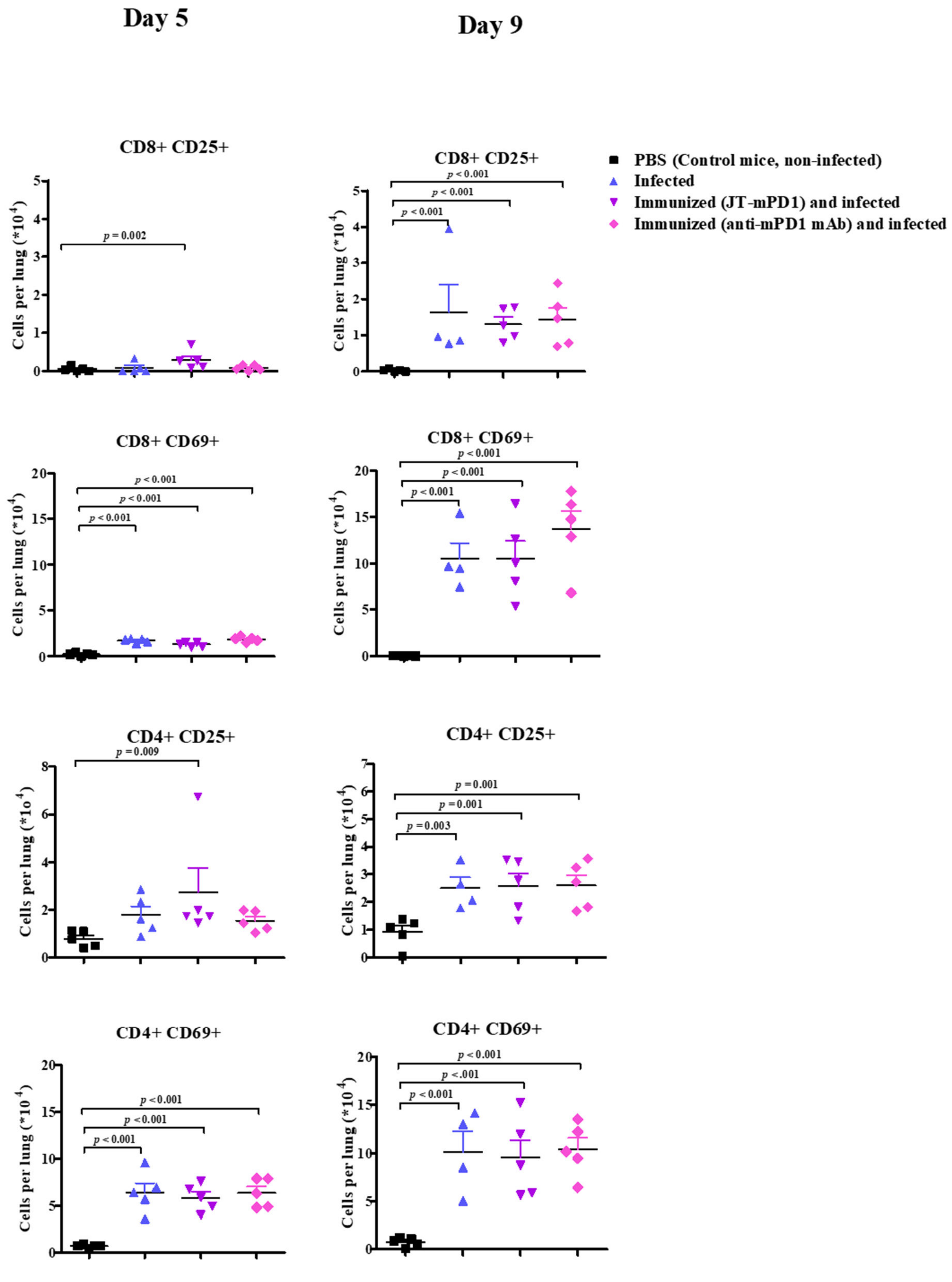
Day 5
Day 9
CD8+ CD25+
CD8+ CD69+
CD4+ CD25+
CD4+ CD69+
Cells per lung (*10^4)
p = 0.002
p < 0.001
p = 0.009
p = 0.001
p = 0.003
p <.001
PBS (Control mice, non-infected)
Infected
Immunized (JT-mPD1) and infected
Immunized (anti-mPD1 mAb) and infected

A

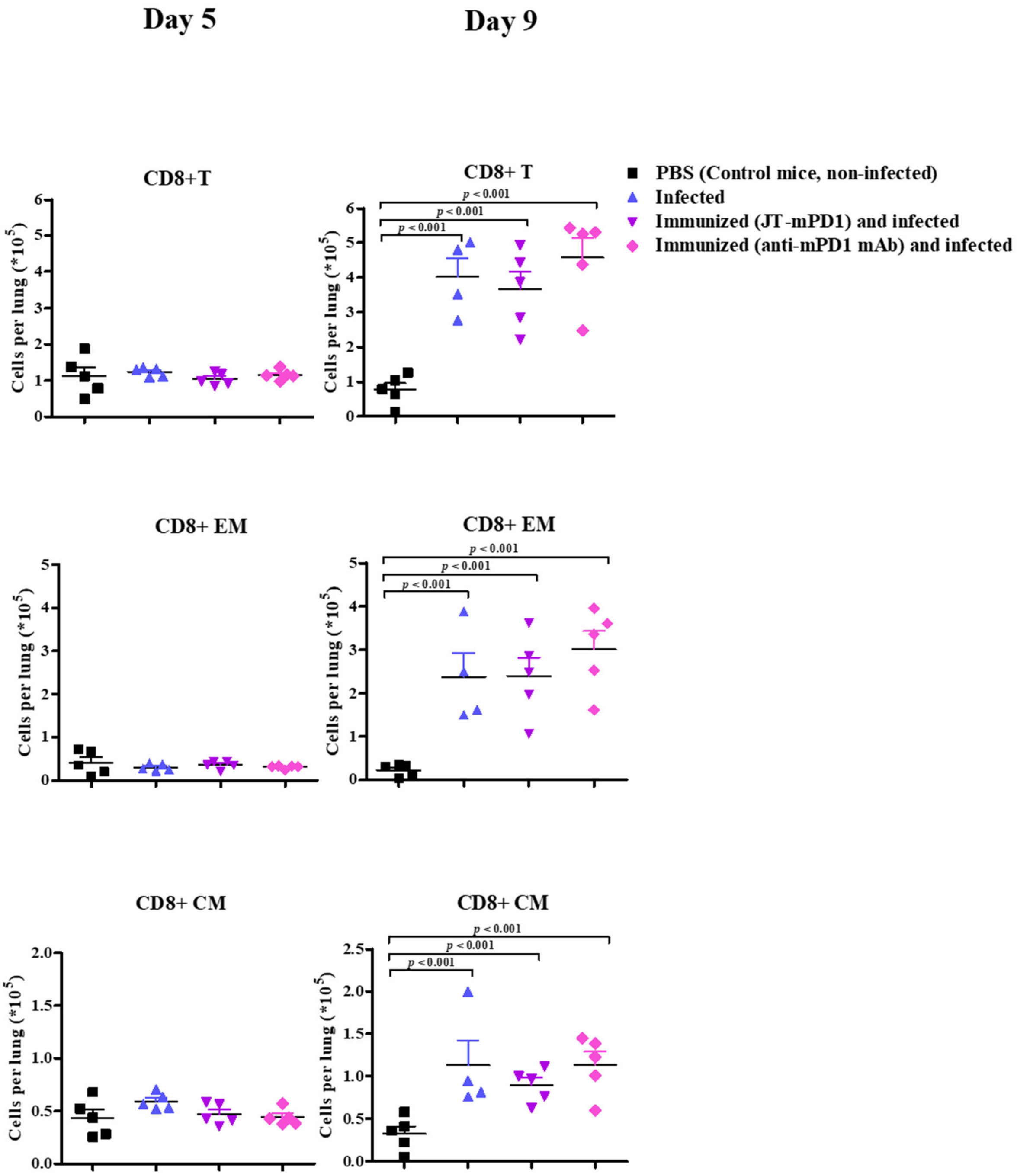

B

Figure S3. Distribution of immune cell profiles. (**A**) Distribution of immune cell profiles in the examined mice measured by flow cytometry in lungs on day 5 and 9 after infection; (**B**) distribution of immune cell profiles in the examined mice measured by flow cytometry in lungs on day 5 and 9 after infection.

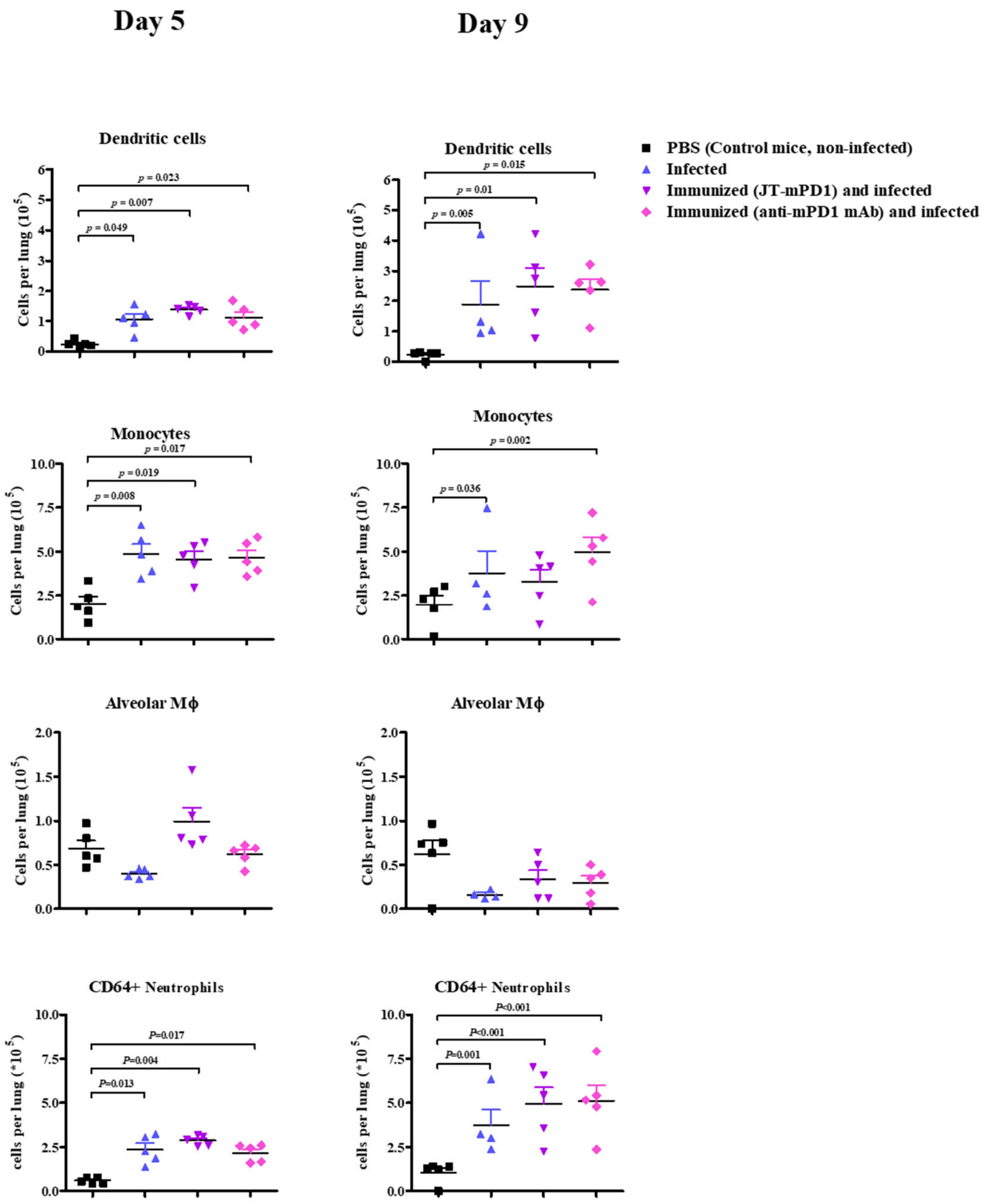

Figure S4. Distribution of myeloid cell profiles in the examined mice measured by flow cytometry in lungs on day 5 and 9 after infection.

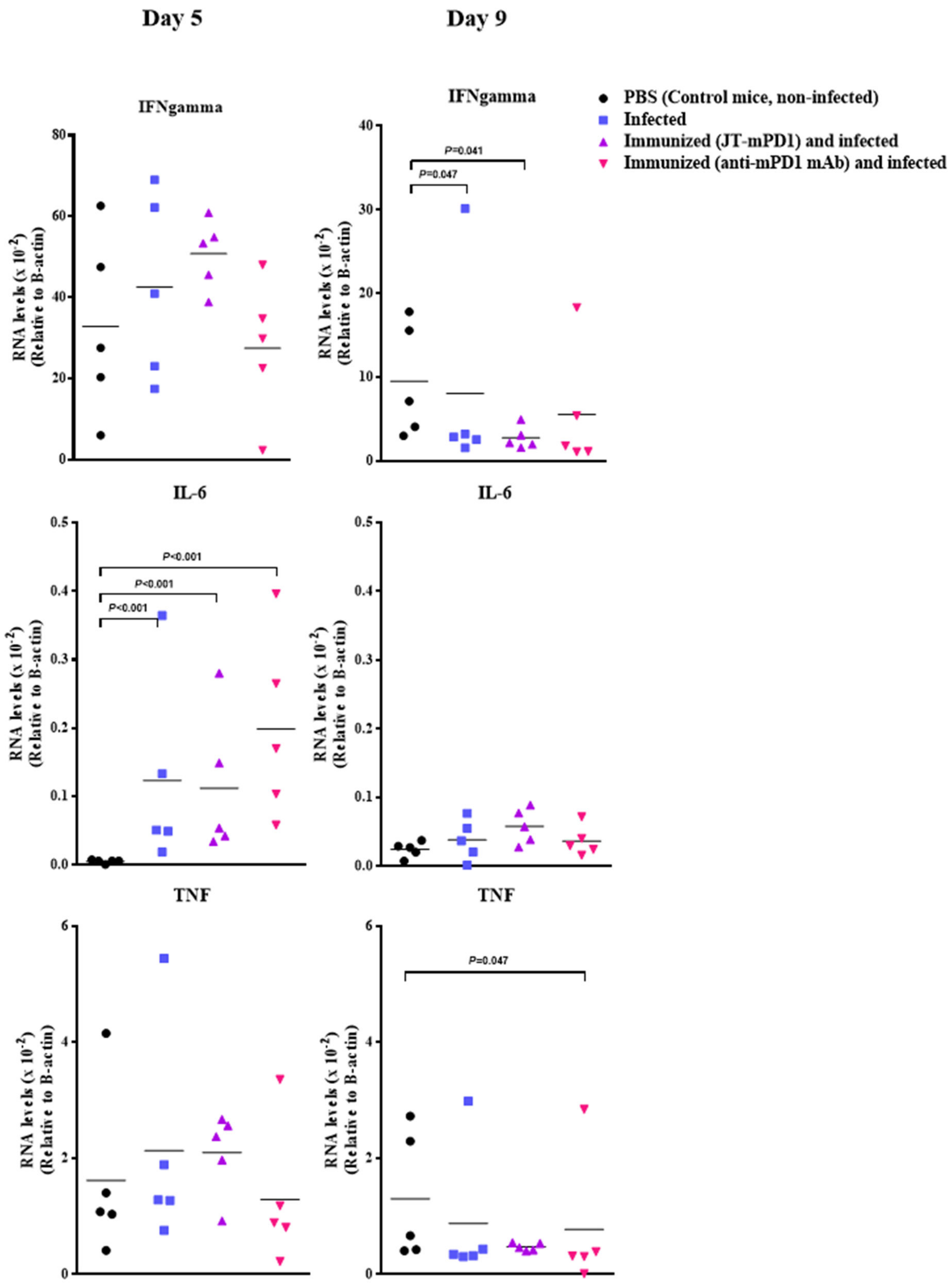

Figure S5. The levels of mRNA expression of IFNgamma, IL-6 and TNF in lungs, measured by RT-PCR.
